# Loneliness and Health Service Utilization among the Rural Elderly in Shandong, China: A Cross-Sectional Study

**DOI:** 10.3390/ijerph15071468

**Published:** 2018-07-11

**Authors:** Jiao Zhang, Lingzhong Xu, Jiajia Li, Long Sun, Gan Ding, Wenzhe Qin, Qian Wang, Jing Zhu, Zihang Yu, Su Xie

**Affiliations:** 1 School of Public Health, Shandong University, Jinan 250012, China; 201614324@mail.sdu.edu.cn (J.Z.); lijiajia@sdu.edu.cn (J.L.); sunlong@sdu.edu.cn (L.S.); dinggan90@163.com (G.D.); qinwenzhe09@163.com (W.Q.); wangqian0519@126.com (Q.W.); zj1176559133@163.com (J.Z.); 13249220350@163.com (Z.Y.); xs340823@163.com (S.X.); 2 Collaborative Innovation Center of Social Risks Governance in Health, School of Public health, Fudan University, Shanghai 200032, China; 3 Key Laboratory of Health Economics and Policy Research, School of Public Health, Shandong University, Jinan 250012, China

**Keywords:** elderly, loneliness, health service utilization

## Abstract

Objectives: To examine the prevalence of loneliness and to explore the association between loneliness and health service utilization among the rural elderly in Shandong Province, China. Methods: A total of 5514 rural people aged 60 and above from Shandong Province, China, were enrolled in this study. Loneliness was used as a binary variable based on a single-item question. Health service utilization was measured by recent two-week physician visits and annual hospitalizations rates. Multiple logistic regression analysis was performed to examine the association between loneliness and health service utilization. Results: The prevalence of loneliness among the rural elderly in Shandong, China, was 25.0%. Loneliness was associated with higher rates of recent two-week physician visits (OR = 1.260, *p* < 0.01) and annual hospitalizations (OR = 1.183, *p* < 0.05). The regression results also showed that self-rated health status and chronic conditions were significant and positively associated with both physician visits and hospitalizations rates. Conclusions: Loneliness had a significant association with higher odds of health service utilization among the elderly. The independent contribution of loneliness on health service utilization was smaller than self-rated health status and chronic conditions. Thus, healthcare policies need to shift from an emphasis on controlling health utilization and cost to a greater focus on enabling lonely older people to get more social support.

## 1. Introduction

A World Health Organization (WHO) report on aging and health pointed out that the proportion of the world’s population over 60 years of age will nearly double, from 12% to 22%, between 2015 and 2050 [1]. Consistent with global trends, China is a rapidly aging society, where 230.86 million (16.7%) people were aged 60 and above in 2016 [2]. By the 2030, this number is expected to rise to over 360 million [3]. However, many older people live longer but do not necessarily have healthier lives. According to another WHO report, approximately 15% of adults aged 60 and over suffer from a mental disorder, including isolation, loss of independence, loneliness, and psychological distress [4].

Loneliness can be particularly important among older adults, for whom decreases in economic resources, increases in impairments, and the deaths of contemporaries can heighten the risk of social isolation and loneliness [5]. A recent study in China indicated that about 28% of older Chinese adults reported feeling lonely [6], which was a public health problem in need of a solution. Many researches have consistently found that loneliness was associated with poor health outcomes, such as high blood pressure [7], cardiovascular disease [8], cognitive decline [9], depression [10], functional decline [11], and mortality [6,11]. Loneliness and related health outcomes could possibly result in higher need for health care or higher health service utilization for the older person.

However, research focusing on the link between loneliness and health service utilization was sparse and was mainly concentrated on the populations of developed countries. In a study from the United States of America (USA) based on two waves of cross-sectional data (2008 and 2012), older adults with chronic loneliness were significantly and positively associated with physician visits [12]. Another longitudinal study from Canada suggested that being lonely was associated with a greater number of physician visits, though the relationship was mediated by health [13]. Another similar finding had been reported in Sweden, where researchers found that frail elders who were lonely used more outpatient services, including more acute visits, compared with those who were not lonely [14]. Nevertheless, Theeke et al. [15] found that the relationship between loneliness and a greater number of physician visits over two years became non-significant when controlling for other confounding factors. In addition, a recent study based on an Asian population showed a significant association between loneliness and lower odds of physician visits among Singaporeans aged 60 years and older [16]. 

The evidence was also mixed as to whether loneliness predicts admission to the hospital. For example, a study in Sweden suggested that elderly people who used inpatient services reported more loneliness than non-users of hospital care [17]. In the research of Gerst-Emerson et al. based on older populations in the USA, however, loneliness was not significantly associated with hospitalization [12]. Therefore, the association between loneliness and health service utilization is still inconclusive. To date, no similar study has been carried out among older people—one of the most vulnerable populations in China—and the relationship between loneliness and health service utilization remains unclear among the Chinese elderly.

To fill the gap in the literature, the aim of this study is to (1) examine the prevalence of loneliness among the rural elderly in Shandong, China, and (2) explore the association between loneliness and health service utilization among older adults.

## 2. Materials and Methods 

### 2.1. Data and Sample

Our data were collected from the 2017 Survey of the Shandong Elderly Family Health Service, which was conducted by Shandong University. We employed stratified multi-stage random sampling method to selected participants: in the first stage, 6 counties were selected from 137 counties as the primary sampling units (PSUs) throughout the eastern, central, and western areas of Shandong Province (which were divided into 3 districts and 3 counties that represented urban and rural areas separately). From each PSU, 18 villages in the rural area and 18 communities in the suburban and urban area were selected as the secondary sampling units (SSUs). In the third stage, based on the roster of the residents by age and the total elderly population of each selected site provided by the local residential committee, an average of 66 individuals were stratified and randomly selected from each SSU making up the total sample. The eligible participants for this survey were those aged 60 or older with local household registrations at the time of the interview. Initially, 7088 elderly individuals were selected and interviewed. Of these, 18 did not complete the survey. In total, 7070 individuals were included in the final sample. About 30 min of face-to-face interviewing were conducted in every participant’s home using a structured questionnaire by a doctoral/master student. To ensure the quality of the survey, first, all students received professional face-to-face interview training before the survey began. Second, the eligible respondents for the survey were willing and able to give a clear answer based on their understanding of the interviewer’s questions. Finally, completed questionnaires were carefully checked by quality supervisors at the end of each day.

This current study focused on respondents in the rural area with a sample of N = 5514. 

### 2.2. Independent Variable

Loneliness was our main independent variable. We assessed loneliness among the elderly using a single question, “I feel lonely”, with four response options: never, rarely, sometimes, and always. Based on these responses, we dichotomized loneliness into those who replied “rarely”, “sometimes”, or “always” (code 1 = lonely) and those who responded “never” (code 2 = not lonely). This single-item self-report measure has been shown to be highly correlated with a multiple-item loneliness scale (the University of California Los Angeles (UCLA) Loneliness Scale); for example, in an empirical report about loneliness among senior citizens, the scores on single-item measures were highly correlated with the UCLA Loneliness Scale (r = 0.72) [18]. Another study about loneliness among senior citizens using meta-analysis [19] showed that among 182 studies, about 40.12% (73/182) used a single-item measure of frequency of loneliness, while 21.98% (40/182) used the UCLA Loneliness Scale. The use of other scales was quite rare (per scale < 7%). In other words, single-item measures have been used extensively in previous research in this field.

### 2.3. Dependent Variable

Health service utilization was measured by two binary variables, namely outpatient service (recent two-week physician visits) and inpatient service (annual hospitalizations). A record of a recent two-week physician visit was obtained by the question: “Have you ever visited a doctor in the past two weeks before survey?”. A record of annual hospitalizations was obtained by the question: “Have you been hospitalized in the last year before survey?”.

### 2.4. Other Covariates

Based on Andersen’s behavioral model [20], we categorized the covariates into three types: predisposing, enabling, and need factors. In the present study, the predisposing factors were age (continuous), gender (male, female), marital status (married, not married/divorced/widowed, or other), and education (no education, primary, junior, or above); the enabling factors were type of health insurance (Urban Employee Basic Medical Insurance (UEBMI), Urban and Rural Residents Basic Medical Insurance (URRBMI), others, or none), living arrangement (live alone, live with another person, or live with three or more people), and personal yearly income (four income quartiles: quartile 1 (Q1) is the poorest and quartile 4 (Q4) is the richest.); and the need factors were self-rated health status (good, moderate, or bad) and chronic health conditions (none, only one, or two or more chronic diseases). We also included a disability measure, using the activities of daily living (ADL) scale [21]. Higher scores from the ADL scale corresponded to greater disabilities in the elderly.

### 2.5. Statistical Analysis

We presented descriptive statistics of the sample by loneliness (lonely vs not lonely), using the *t* test to examine statistical significance in continuous variables and a chi-square test for categorical variables. The chi-square test was also used to compare health service utilization between lonely and not lonely elderly. To further explore the association between loneliness and health service utilization (outpatient services and inpatient services), multivariate logistic regression was performed to assess the explanatory variables of using of outpatient services and inpatient services separately. The odds ratio (OR) with corresponding 95% confidence intervals (95% CI) were used as a measure of association. All data were analyzed using SPSS 24.0 (IBM Crop, Armonk, NY, USA). *p*-values < 0.05 were considered to be statistically significant.

## 3. Results

### 3.1. Respondents’ Characteristics by Loneliness

Characteristics of the sample, as well as the prevalence of loneliness among the rural elderly, are shown in Table 1. Generally speaking, the majority of the rural elderly were female (57.1%), married (80.4%), having primary education (41.3%), URRBMI (93.1%), living with another person (65.3%), having good self-rated health status (52.2%), and having one chronic disease (36.1%). In a total of 5514 rural elderly, 1381 (25.0%) reported feeling lonely, of which females accounted for 58.1%. The lonely elderly’s average age was 70.1 ± 2.2 and their ADL score was 15.5 ± 4.6. In addition, age (*p* < 0.05), marriage status (*p* < 0.001), education (*p* < 0.05), living arrangement (*p* < 0.001), income (*p* < 0.001), self-rated health status (*p* < 0.001), number of chronic conditions (*p* < 0.001), and ADL (*p* < 0.001) were significantly different between rural lonely and not lonely elderly.

### 3.2. Comparison of Health Service Utilization between Lonely and Not Lonely Elderly

Table 2 shows the using of health services among the rural elderly. From the result, significant differences between the two groups were found regarding the using of outpatient services and inpatient services. Overall, the prevalence of recent two-week physician visits and annual hospitalizations were 21.3% and 17.6% in the rural elderly, respectively. When comparing the using of outpatient services between the two groups, the group who reported feeling lonely had a significantly higher physician visit rate (26.1%) than that of “not lonely” group (19.8%) (*p* < 0.001). The same result was also found for using of inpatient services; the annual hospitalizations rate among the lonely elderly (21.9%) was higher than that of “not lonely” elderly (16.2%) (*p* < 0.001).

### 3.3. Associations between Loneliness, Related Influential Factors, and Health Service Utilization

Table 3 presents multivariate logistic regression analyses that showed the association between health service utilization, loneliness, and other factors. Even after controlling for the effects of other covariates, including sociodemographic and health-related variables, there was a significant association between loneliness and higher odds of utilizing physician visits (OR = 1.260, *p* < 0.01) and hospitalizations (OR = 1.183, *p* < 0.05), compared with the elderly who were not lonely. For the predisposing factors, the result showed that the female elderly were likely to experience more physician visits (OR = 1.178, *p* < 0.05) but fewer hospitalizations (OR = 0.824, *p* < 0.05), compared with the male elderly. Being single was associated with higher odds of utilizing physician visits (OR = 1.393, *p* < 0.05) compared with those who were married. Among the enabling factors, lower income groups had different patterns of using of outpatient services and inpatient services. For instance, compared with the highest income group, group Q1 was significantly associated with lower odds of utilizing physician visits (OR = 0.761, *p* < 0.01) but was significantly associated with 24.7% higher odds of hospitalizations (*p* < 0.05). The need factors were strong determinants of health service utilization. Self-rated “moderate” or “bad” health statuses were significantly correlated with both outpatient services (OR = 1.217, *p* < 0.05; OR = 2.014, *p* < 0.001, respectively) and inpatient services (OR = 1.597, *p* < 0.001; OR = 2.463, *p* < 0.001, respectively). As regards chronic conditions, compared with those elderly without any disease, having one chronic disease was significantly associated with more than triple odds of utilizing outpatient services (OR = 3.336, *p* < 0.001) and double odds of utilizing inpatient service (OR = 2.019, *p* < 0.001), while for those with at least two chronic diseases, the odds of both going for physician visits and being hospitalized were more than quadrupled (*p* < 0.001). The results showed that type of health insurance and the ADL score had no significant association with any health service in this study.

## 4. Discussion

The increasing literature based on western or Asian countries has indicated that loneliness is associated with higher or lower health care consumption in older adults. Our study extended this research to examine the prevalence of loneliness and its relationship with health service utilization among older adults in China, which has the largest number of the older people in the world. The results showed that among the rural older people of Shandong, China, 25% reported feeling lonely. It was lower than the reported loneliness rate of 29.6% in 2000 [22], 28% in 2008 [6], and 33.1% in 2011 [23] among the senior citizens from the nationally representative sample. The prevalence of loneliness may vary widely due to different settings and applied measures in each study. Another possible explanation is that increasing young people began to pay attention to the severe loneliness among the elderly after “Family members should be concerned about the spiritual needs of the elderly and must not ignore the elderly. Family members living separately from the elderly should often visit or greet the elderly” was listed in the revised law of the People’s Republic of China on the Protection of the Rights and Interests of the Elderly [24]. “Spiritual support” for the elderly was first written into this law, which has been implemented since 1 July 2013. Further research still needs to be conducted to explain this phenomenon.

The hypothesis that loneliness is associated with increased health service utilization was supported by the data. We found that after taking into account socio-demographics and health status, loneliness was significantly associated with 26.0% higher odds of physician visits, compared with being not lonely. This result was consistent with previous research in western countries [12,13,14,25]. For hospitalization, this study showed that the elderly with feeling of loneliness tended to have more frequency of hospitalizations, which had some differences with previous findings. For example, one study based on adults (aged 45 and above years) in Canada found that being lonely was not associated with higher odds of admission to the hospital, except being re-hospitalized [13]. Also, it was not similar to another finding by Molloy et al., who found that among Irish elders, loneliness was significantly associated with emergency hospitalization but not with planned hospitalization [26]. 

Causal reasons why the lonely elderly are utilizing both more physician visits and hospitalizations were beyond the scope of this cross-sectional study. However, the prevalence of a special doctor–patient relationship among lonely elders, which has been supported in many previous studies, may explain this phenomenon. Socially isolated persons, especially the elderly, were more likely to seek social support and interaction with people rather than solely medical treatment, and medical doctors possibly fulfilled a social role for those who needed someone with whom to talk [12,27,28,29]. Other explanations were also possible but deserved further exploration. For example, lonely individuals may be less able to manage their health effectively because of behavioral, emotional, and cognitive regulatory deficits [30], which could negatively affect one’s recovery from illness or make it worse and lead to more physician visits or hospitalizations. In an application of this hypothesis, Pitkala et al. [31] found that social intervention reduced lonely older adults’ physician visits and lengths of stay in the hospital. However, they did not show that the intervention changed the elders’ level of loneliness; therefore, further studies need to test this hypothesis. 

Furthermore, this study supported previous research indicating that, whether subjective or objective, health statuses were significantly associated with the use of health services [32,33,34]. As the results shown, having at least two chronic diseases was significantly associated with more than quadrupled odds of going for physician visits and being hospitalized, compared with those without any disease. According to a population-based study, the impact of chronic diseases on health services utilization was not a simple or direct relationship and may be mediated through health status [35]. This present study cannot explain this problem. However, what our study can determine was that after controlling for the strong effects of chronic diseases, loneliness still predicted more use of health services among the elderly, which has important policy implications. Strategies to cope with health utilization and cost for older adults might need to emphasize enabling them to get more intergenerational relationships and social support. 

The present study has several limitations. First, this is a cross-sectional study, and determining cause and effect is beyond its capability. Therefore, it cannot be conclude that loneliness causes elders’ health service use behaviors. Second, there may be more confounding factors than those available for consideration in this study. Finally, we did not tease apart the type of physician visits or hospitalizations in the current study, which will be analyzed explicitly in the follow-up study. 

## 5. Conclusions

The prevalence of loneliness among the rural elderly in Shandong, China was 25.0%. Loneliness had a significant association with higher odds of health service utilization among the elderly. The independent contribution of loneliness on health service utilization was smaller than self-rated health status and chronic conditions. Healthcare policies needed to shift from an emphasis on controlling health service utilization and cost to a greater focus on enabling lonely older people to get more social support.

## Figures and Tables

**Table 1:** Characteristics of the elderly by loneliness.

Characteristic	Total Sample	Lonely	Not Lonely	*p*
**Observations *n* (%)**	5514 (100)	1381 (25.0)	4133 (75.0)	
**Predisposing factors**				
**Age mean ± SD**	69.7 ± 6.5	70.2 ± 6.6	69.6 ± 6.5	0.003
**Gender *n* (%)**				0.394
Male	2366 (42.9)	579 (41.9)	1787 (43.2)	
Female	3148 (57.1)	802 (58.1)	2346 (56.8)	
**Marriage status *n* (%)**				<0.001
Married	4433 (80.4)	923 (66.8)	3510 (84.9)	
Single ^a^	1081 (19.6)	458 (33.2)	623 (15.1)	
**Education *n* (%)**				0.001
No education	2164 (39.2)	602 (43.6)	1562 (37.8)	
Primary	2277 (41.3)	522 (37.8)	1755 (42.5)	
Junior or above	1073 (19.5)	257 (18.6)	816 (19.7)	
**Enabling factors**				
**Type of health insurance ^b^**				0.231
UEBMI	265 (4.8)	76 (5.5)	189 (4.6)	
URRBMI	3824 (93.1)	1272 (92.1)	3859 (93.4)	
Others	39 (0.7)	8 (0.6)	31 (0.8)	
None	79 (1.4)	25 (1.8)	54 (1.3)	
**Living arrangement *n* (%)**				<0.001
1	775 (14.1)	365 (26.4)	410 (9.9)	
2	3600 (65.3)	812 (58.8)	2788 (67.5)	
≥3	1139 (20.7)	204 (14.8)	935 (22.6)	
**Income *n* (%) ^c^**				<0.001
Q1	1590 (28.8)	478 (34.6)	1112 (26.9)	
Q2	1191 (21.6)	318 (23.0)	873 (21.1)	
Q3	1362 (24.7)	310 (22.4)	1052 (25.5)	
Q4	1371 (24.9)	275 (19.9)	1096 (26.5)	
**Need factors**				
**Self-rated health status *n* (%)**				<0.001
Good	2878 (52.2)	575 (41.6)	2303 (55.7)	
Moderate	1508 (27.3)	408 (29.5)	1100 (26.6)	
Bad	1128 (20.5)	398 (28.8)	730 (17.7)	
**Number of chronic conditions *n* (%)**				<0.001
None	1718 (31.2)	332 (24.0)	1386 (33.5)	
1	1993 (36.1)	514 (37.2)	1479 (35.8)	
≥2	1803 (32.7)	535 (38.7)	1268 (30.7)	
**ADL mean ± SD**	15.5 ± 4.6	16.3 ± 5.9	15.2 ± 4.0	<0.001

^a^ Single: not married/divorced/widowed or others; ^b^ UEBMI: Urban Employee Basic Medical Insurance; URRBMI: Urban and Rural Residents Basic Medical Insurance; ^c^ Income: personal yearly income; SD: standard deviation; and ADL: activities of daily living.

**Table 2:** Comparison of health service utilization between lonely and not lonely rural elderly in Shandong, China.

Health Service Utilization	Total	Lonely (%)	Not Lonely (%)	*p*
**Outpatient service**				<0.001
Physician visits	1177 (21.3)	360 (26.1)	817 (19.8)	
No use	4337 (78.7)	1021 (73.9)	3316 (80.2)	
**Inpatient service**				<0.001
Admission	972 (17.6)	302 (21.9)	670 (16.2)	
No use	4542 (82.4)	1079 (78.1)	3463 (83.8)	

**Table 3:** Association of loneliness and using of health service in Shandong, China.

Variable	Model I (Outpatient Service)			Model II (Inpatient Service)		
	Users	*p*	OR (95% CI)	Users	*p*	OR (95% CI)
**Loneliness**						
Lonely	360 (30.6)	0.003	1.260 (1.079, 1.472)	302 (31.1)	0.047	1.183 (1.002, 1.397)
Not lonely (ref)	817 (69.4)			670 (68.9)		
**Predisposing factors**						
**Age mean ± SD**	69.5 ± 6.0	0.050	0.988 (0.977, 1.000)	70.2 ± 6.4	0.127	1.010 (0.997, 1.022)
**Gender *n* (%)**						
Male (ref)	443 (37.6)			418 (43.0)		
Female	734 (62.4)	0.034	1.178 (1.012, 1.372)	554 (57.0)	0.020	0.824 (0.700, 0.970)
**Marriage status *n* (%)**						
Married (ref)	945 (80.3)			784 (80.7)		
Single ^a^	232 (19.7)	0.019	1.393 (1.056, 1.837)	188 (19.3)	0.628	0.927 (0.682, 1.260)
**Education *n* (%)**						
No education	480 (40.8)	0.350	1.107 (0.895, 1.370)	415 (42,7)	0.675	0.953 (0.763, 1.192)
Primary	499 (42.4)	0.067	1.202 (0.987, 1.465)	370 (38.1)	0.078	0.829 (0.673, 1.021)
Junior or above (ref)	198 (16.8)			187 (19.2)		
**Enabling factors**						
**Type of insurance ^b^**						
UEBMI (ref)	52 (4.4)			47 (4.8)		
URRBMI	1100 (93.5)	0.348	1.172 (0.841, 1.634)	909 (93.5)	0.988	1.003 (0.707, 1.421)
Others	11 (0.9)	0.094	1.967 (0.891, 4.341)	5 (0.5)	0.748	0.846 (0.306, 2.342)
None	14 (1.2)	0.946	0.977 (0.496, 1.923)	11 (1.1)	0.636	0.837 (0.400, 1.751)
**Living arrangement *n* (%)**						
1	153 (13.0)	0.032	0.712 (0.521, 0.971)	135 (13.9)	0.846	0.967 (0.689, 1.358)
2	808 (68.6)	0.009	1.281(1.063, 1.544)	657 (67.6)	0.366	1.096 (0.898, 1.339)
≥3 (ref)	216 (18.4)			180 (18.5)		
**Income ^c^*n* (%)**						
Q1	338 (28.7)	0.007	0.761 (0.624, 0.929)	316 (32.5)	0.050	1.247 (1.000, 1.555)
Q2	280 (23.8)	0.386	0.913 (0.744, 1.121)	218 (22.4)	0.244	1.148 (0.910, 1.449)
Q3	274 (23.3)	0.008	0.761 (0.623, 0.930)	251 (25.8)	0.067	1.231 (0.986, 1.538)
Q4 (ref)	285 (24.2)			187 (19.2)		
**Need factors**						
**Self-rated health status *n* (%)**						
Good (ref)	434 (36.9)			304 (31.3)		
Moderate	347 (29.5)	0.021	1.217 (1.030, 1.438)	308 (31.7)	<0.001	1.597 (1.332, 1.914)
Bad	396 (33.6)	<0.001	2.014 (1.685, 2.408)	360 (37.0)	<0.001	2.463 (2.032, 2.985)
**Number of chronic conditions *n* (%)**						
None (ref)	128 (10.9)			120 (12.3)		
1	458 (38.9)	<0.001	3.336 (2.695, 4.131)	309 (31.8)	<0.001	2.019 (1.607, 2.536)
≥2	591 (50.2)	<0.001	4.901 (3.943, 6.093)	543 (55.9)	<0.001	4.119 (3.292, 5.155)
**ADL mean ± SD**	15.8 ± 4.9	0.104	0.988 (0.973, 1.003)	16.3 ± 5.7	0.801	1.002 (0.988, 1.016)

^a^ Single: not married/divorced/widowed or others; ^b^ UEBMI: Urban Employee Basic Medical Insurance; URRBMI: Urban and Rural Residents Basic Medical Insurance; ^c^ Income: personal yearly income; OR: odds ratio; SD standard deviation; and ADL: activities of daily living.

## Abbreviations

WHO	World Health Organization
PSU	Primary Sampling Unit
SSU	Secondary Sampling Unit
UCLA	University of California Los Angeles Loneliness Scale
UEBMI	Urban Employee Basic Medical Insurance
URRBMI	Urban and Rural Residents Basic Insurance
ADL	Activities of Daily Living
Q1–Q4	Quartile 1–Quartile 4
